# Morphological and DNA-based description of *Trichophoromyia peixotoi* n. sp. (Diptera: Psychodidae), a new sand fly species from the Brazilian Amazon

**DOI:** 10.1186/s13071-023-05850-w

**Published:** 2023-07-19

**Authors:** Bruno Leite Rodrigues, Israel de Souza Pinto, Eunice Aparecida Bianchi Galati

**Affiliations:** 1 Programa de Pós-Graduação em Saúde Pública, Faculdade de Saúde Pública, Universidade de São Paulo (FSP/USP), São Paulo, SP Brazil; 2 Instituto Federal de Educação, Ciência e Tecnologia do Pará (IFPA), Itaituba, PA Brazil; 3 Departamento de Epidemiologia, Faculdade de Saúde Pública, Universidade de São Paulo (FSP/USP), São Paulo, SP Brazil

**Keywords:** Phlebotominae, Integrative taxonomy, DNA barcoding, *COI*

## Abstract

**Background:**

Phlebotomine sand flies of the genus *Trichophoromyia* Barretto, 1962 are of great relevance to public health as vectors of *Leishmania* protozoans. A new phlebotomine species named *Trichophoromyia peixotoi* n. sp. is here described based on both male morphology and *COI* DNA barcodes.

**Methods:**

The sand fly specimens were collected in the Parque Nacional da Amazônia (PNA), situated in the municipality of Itaituba, state of Pará, Brazil. Morphological description was done based on 10 male specimens. Five specimens were DNA barcoded for the *COI* gene.

**Results:**

The morphological and molecular analyses allowed the delimitation of this new species from others of *Trichophoromyia*. *Trichophoromyia peixotoi* n. sp. is closely related to other species with aedeagal ducts > 4 times the length of the sperm pump, from which it may be distinguished by the gonocoxite bristles and paramere shape.

**Conclusions:**

The description of *T. peixotoi* n. sp. brings the number of species of *Trichophoromyia* to 45, including 24 for Brazil. The integrative taxonomy effort through the analysis of *COI* barcodes proved to be effective in the species delimitation of some *Trichophoromyia* spp.

**Graphical Abstract:**

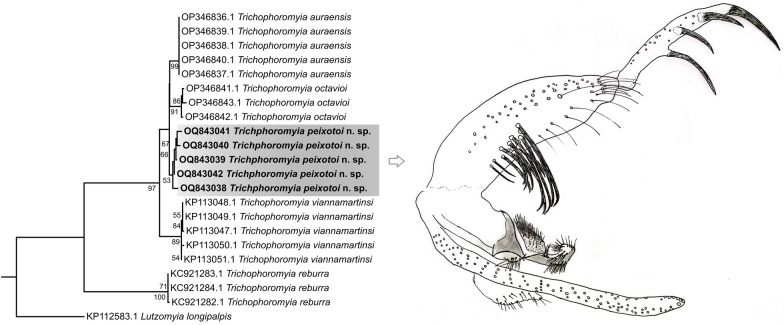

## Background

 Phlebotomine sand flies are important vectors of several pathogens of relevance for public health, including viruses, bacteria, and *Leishmania* protozoans [1]. The Phlebotominae subfamily comprises about 1060 species distributed worldwide [2], which are sorted into several tribes, subtribes, and genera according to Galati [3, 4]. One of the Neotropical subtribes—Psychodopygina Galati, 2003—is subdivided into seven genera, most of them with vector species of cutaneous leishmaniasis agents, including *Trichophoromyia* Barretto, 1962 [5].

The genus *Trichophoromyia* has been gaining evidence for the control of leishmaniasis, as some of its species have been incriminated as biological vectors and others are suspected vectors [6]. Currently, there are 44 nominal species described, 23 of them occurring in Brazil [2, 7]. Most of its species occur in the Amazon biome, including forest fragments or anthropized environments [7]. These sand flies are usually of medium size and brown or dark brown in colour; both sexes have Newstead’s sensilla on the second palpal segment, while males present terminalia equivalent to or longer than the length of the thorax, and females with spermathecae presenting 25 or more rings, the apical one frequently three or more times longer than the preapical [8].

Most of the taxonomic information on this genus is based on the adult male morphology, as only one species has its immature forms described [9], and few females can be truly distinguished [8]. Therefore, the *Trichophoromyia* taxonomy has many gaps and seems to be tricky. Thus, the use of integrative taxonomy can help by providing new lines of evidence for the delimitation, description of new species, and classification proposals [10]. The sequencing and analysis of molecular markers is one of these additional approaches, and can help to improve the taxonomy and systematics of sand flies [11, 12]. For Neotropical sand flies, DNA barcoding fragments of the cytochrome *c* oxidase subunit I (*COI*) gene [13] supported the description of females of *Psathyromyia pradobarrientosi* (Le Pont, Matias, Martinez & Dujardin, 2004) [14], but it had not been used until then to describe new sand fly taxa in the Americas. Recently, *COI* barcodes have been proposed as diagnoses for descriptions of new species, which might somewhat speed up the laborious process of describing new taxa [15]. However, this type of approach has proven insufficient and unstable for describing new species [16, 17], although it can be used as an additional line of evidence to support the description of new taxa.

A new sand fly species—*Trichophoromyia peixotoi* n. sp.—collected in the Parque Nacional da Amazônia (PNA), located in the Brazilian Amazon, is herein described using morphology and *COI* DNA sequences.

## Methods

### Sample collection

Sand flies were collected between 7 July and 11 November 2022 in the PNA, situated in the municipality of Itaituba, state of Pará, Brazil, bordering the state of Amazonas, with a hot and humid equatorial climate. Its predominant vegetation is the dense ombrophilous forest, typical of the Amazon biome, with numerous trees that can reach 40 m in height. In the boundaries of the eastern region of the PNA—where the sand fly collections were carried out—there is a significant increase in phytophysiognomic characteristics of anthropic action, such as pasture and secondary vegetation with palm trees [18]. Sand fly collections were performed under authorization for activities with scientific purpose of the Brazilian *Sistema de Autorização e Informação em Biodiversidade* (SISBIO) for the capture of zoological material (no. 81991-2).

For sample collection, Centers for Disease Control and Prevention (CDC)-type light traps were installed in two different vertical strata, 1 m above the ground, and in the canopy of trees, about 15 m in height. The traps were installed within the forest, close to the Trans-Amazonian Highway (BR 230), and operated overnight from 7 pm to 7 am of the next day. The three collection sites were set near the Tracoá and Uruá bases of the PNA: Trilha da Capelinha 01 (4°37′21.8″S; 56°23′22.3″W), Trilha da Capelinha 02 (4°36′41.5″S; 56°23′25.3″W), and Trilha de Monitoramento 03 (4°28′22.0″S; 56°17′12.1″W). The collections of sites 01 and 02 were done at the ground level, while those of site 03 were from the canopy.

### Morphology

The description of this new species is based on 10 male specimens. Seven of them had the legs dissected and stored in microtubes for later DNA extraction, while the rest of the body was used for morphological description. Sand flies were clarified according to Forattini [19], and then slide-mounted in Enecê resin [20] or Canada balsam.

The terminology of morphological characters follows Galati et al. [21]. The structures were measured using the Zen version 4.7 program, with images taken with an Axiocam 105 color (Carl Zeiss MicroImaging GmbH, Jena, Germany) coupled to an optical microscope. All drawings were made in pencil using an Olympus camera lucida attached to a microscope.

All measurements are given in micrometers for the holotype, and the mean or range of countable characters, the standard deviation, and the number of analysed specimens for each structure are included in brackets.

### Amplification and data analysis of *COI* DNA barcoding

Total DNA was extracted from legs. For this, plastic pistils were used to macerate the legs in a 1.5 ml microtube with Digsol Buffer (ethylenediaminetetraacetic acid [EDTA] 20 mM, Tris–HCl 50 mM, NaCl 117 mM; sodium dodecyl sulfate [SDS] 1%), and 2 μl of proteinase K at 20 mg/ml. After incubation (55 °C overnight), ammonium acetate (4 M) was added to each tube, vortexed, and then centrifuged for 15 min at 14,000 rpm and 10 °C. The supernatants were washed using absolute and 70°GL alcohol. After drying, 20 μl of TE buffer was added to dissolve the pellet, which was kept at −20 °C until use.

For DNA amplification, the PCR Master Mix (Invitrogen, Thermo Scientific™) was used, following the manufacturer’s instructions, and the primers LCO1490 (5′-GGTCAACAAATCATAAAGATATTGG-3′) and HCO2198 (5′-TAAACTTCAGGGTGACCAAAAAATCA-3′) [22] under the following conditions: an initial denaturation of 95 °C for 2 min; followed by 35 cycles of 95 °C for 1 min, 54 °C for 1 min and 72 °C for 1 min 30 s; and a final extension of 72 °C for 10 min. Samples were checked by electrophoresis using 1% agarose gels stained with GelRed (Biotium, Inc.), and all positive reactions that gave bands of the expected molecular size were sent to ACTGene Análises Moleculares (Brazil) for purification and direct sequencing of the PCR products in both directions (forward and reverse).

Initially, SeqTrace version 0.9.0 (http://seqtrace.googlecode.com/, [23]) was used to manually check the electropherograms, remove primer sequences, and assemble consensus sequences, which were submitted to the National Center for Biotechnology Information (NCBI) GenBank [24], and have been assigned accession numbers OQ843038–OQ843042.

By way of comparison, we constructed a dataset with other publicly available *Trichophoromyia* species. The sequence alignment was done using MUSCLE [25] implemented in MEGA v7 software [26]. Pairwise genetic distances for both maximum intraspecific and minimum interspecific (nearest-neighbour, NN) were generated in the BOLD Systems environment (available at https://boldsystems.org/) using the Barcode Gap Analysis tool and the Kimura 2-parameter (K2P) model. In order to check the clustering pattern of *Trichophoromyia* sequences, the consensus alignment was used to generate a maximum likelihood (ML) phylogenetic tree in the IQ-TREE software [27]. For this, we used the automatic model selection, and 10,000 ultrafast bootstrap pseudoreplicates in the IQ-TREE web server (http://iqtree.cibiv.univie.ac.at/ [28]). A sequence of *Lutzomyia longipalpis* (Lutz & Neiva, 1912) (KP112583.1) was included as an outgroup to root the ML tree. Tree visualization and editing were done in the iTOL v5 web server (https://itol.embl.de [29]).

The DNA barcode sequences were also identified at the molecular operational taxonomic unit (MOTU) level, which are groups of specimens based on their molecular similarity at a given molecular marker [30]. For this, we employed the automatic barcode gap discovery (ABGD) and refined single linkage (RESL) methods [31, 32]. The ABGD analysis (available at https://bioinfo.mnhn.fr/abi/public/abgd/abgdweb.html) was run using the K2P model, and the parameters Pmin = 0.005, Pmax = 0.1, and X = 1.5. For ABGD, the recursive partitions generated with the prior intraspecific divergence of 0.013 were considered. RESL analysis was run inside the BOLD environment using the ‘cluster sequences’ tool and default parameter.

## Results

Family Psychodidae Newman, 1834

Subfamily Phlebotominae Rondani & Berté, in Rondani 1840

Genus *Trichophoromyia* Barretto, 1962

**Fig. 1:**
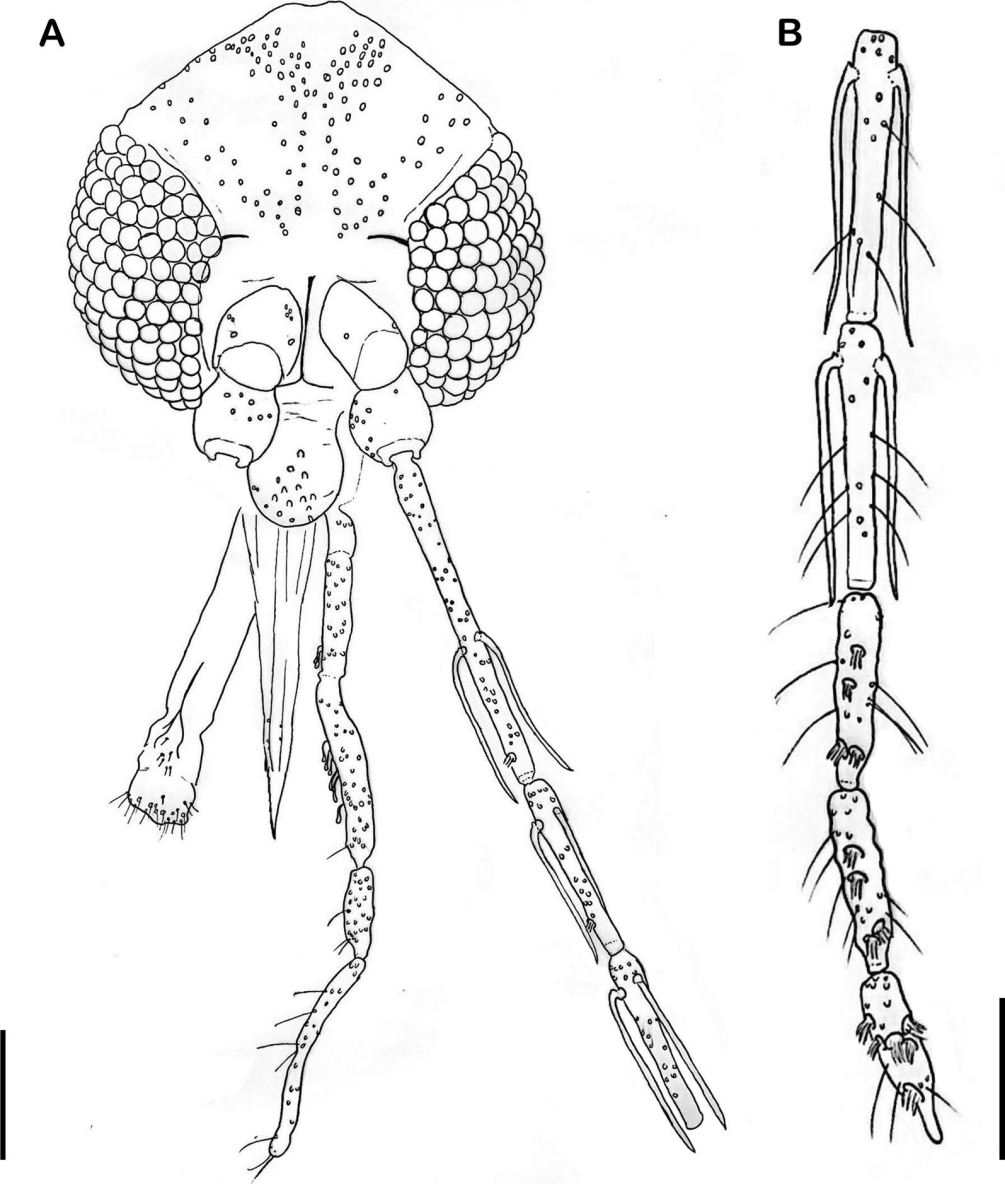
*Trichophoromyia peixotoi* n. sp. holotype: **A** Head, dorsal view (scale bar: 100 μm). **B** Flagellomeres FX–FXIV (scale bar: 50 μm)

**Fig. 2:**
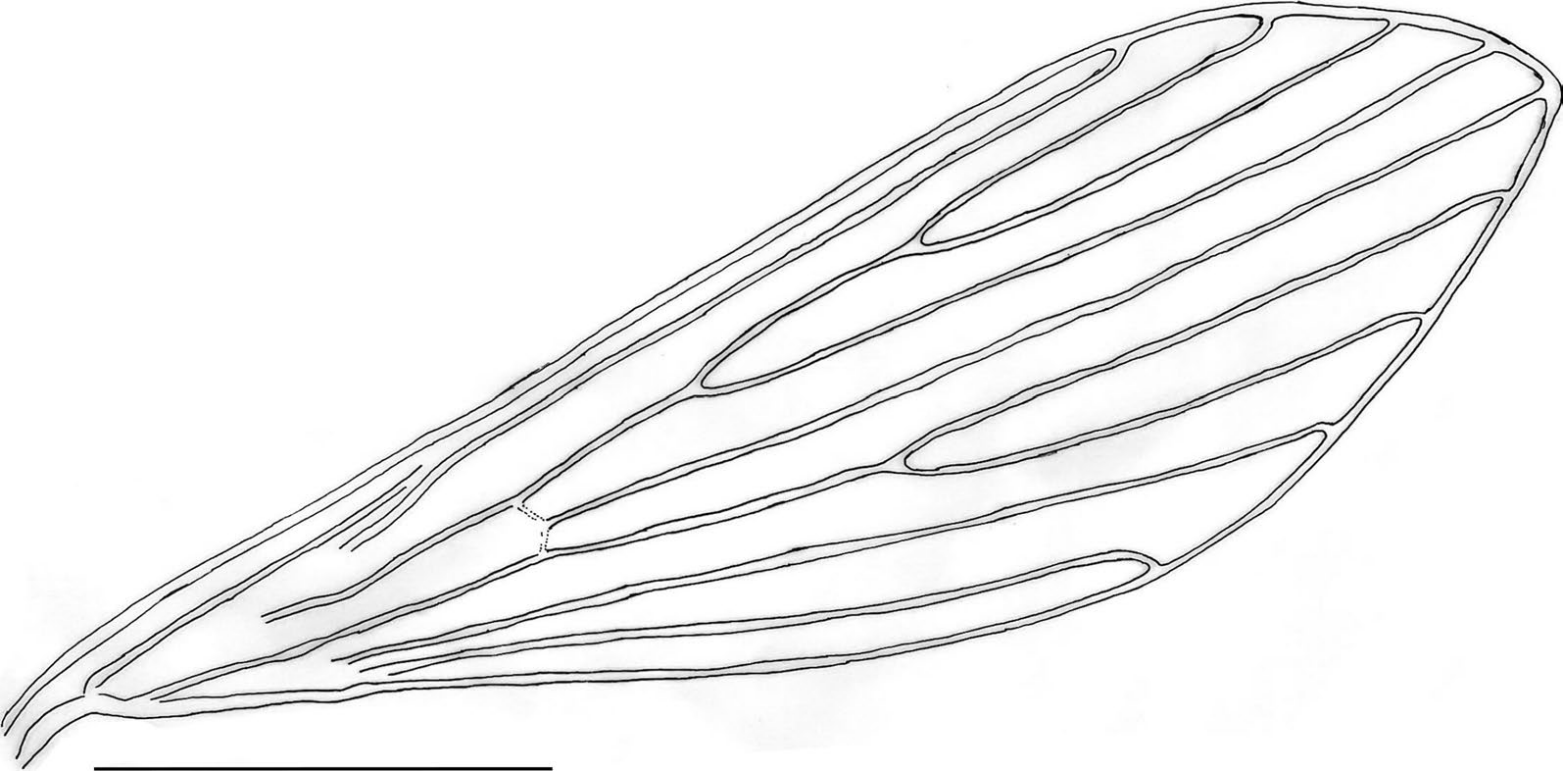
Wing of *Trichophoromyia peixotoi* n. sp. holotype (scale bar: 500 μm)

**Fig. 3:**
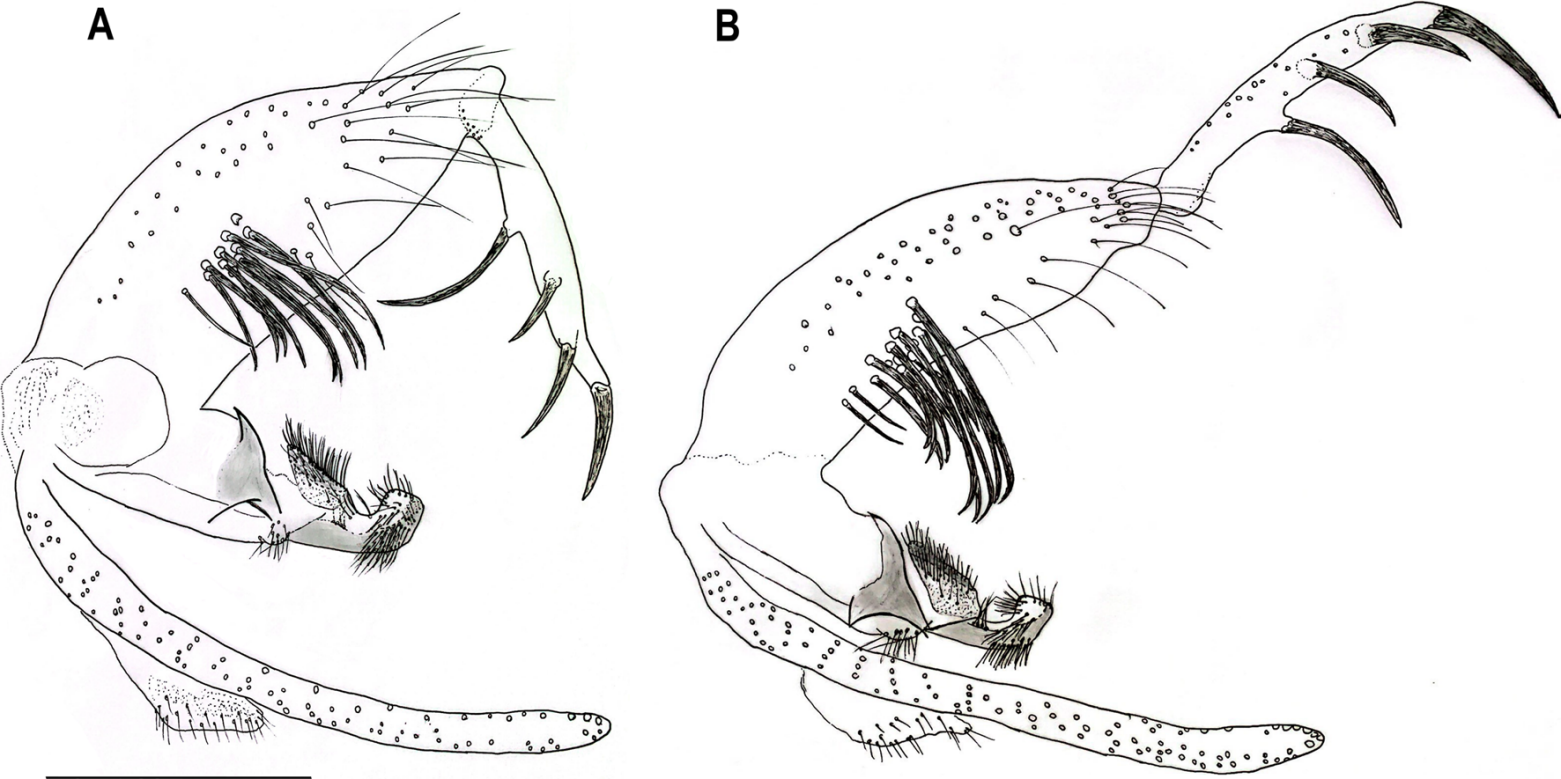
Lateral view of male terminalia of the holotype (**A**) and the paratype M2 (E-16395) (**B**) of *Trichophoromyia peixotoi* n. sp. (scale bar: 200 μm)

**Fig. 4:**
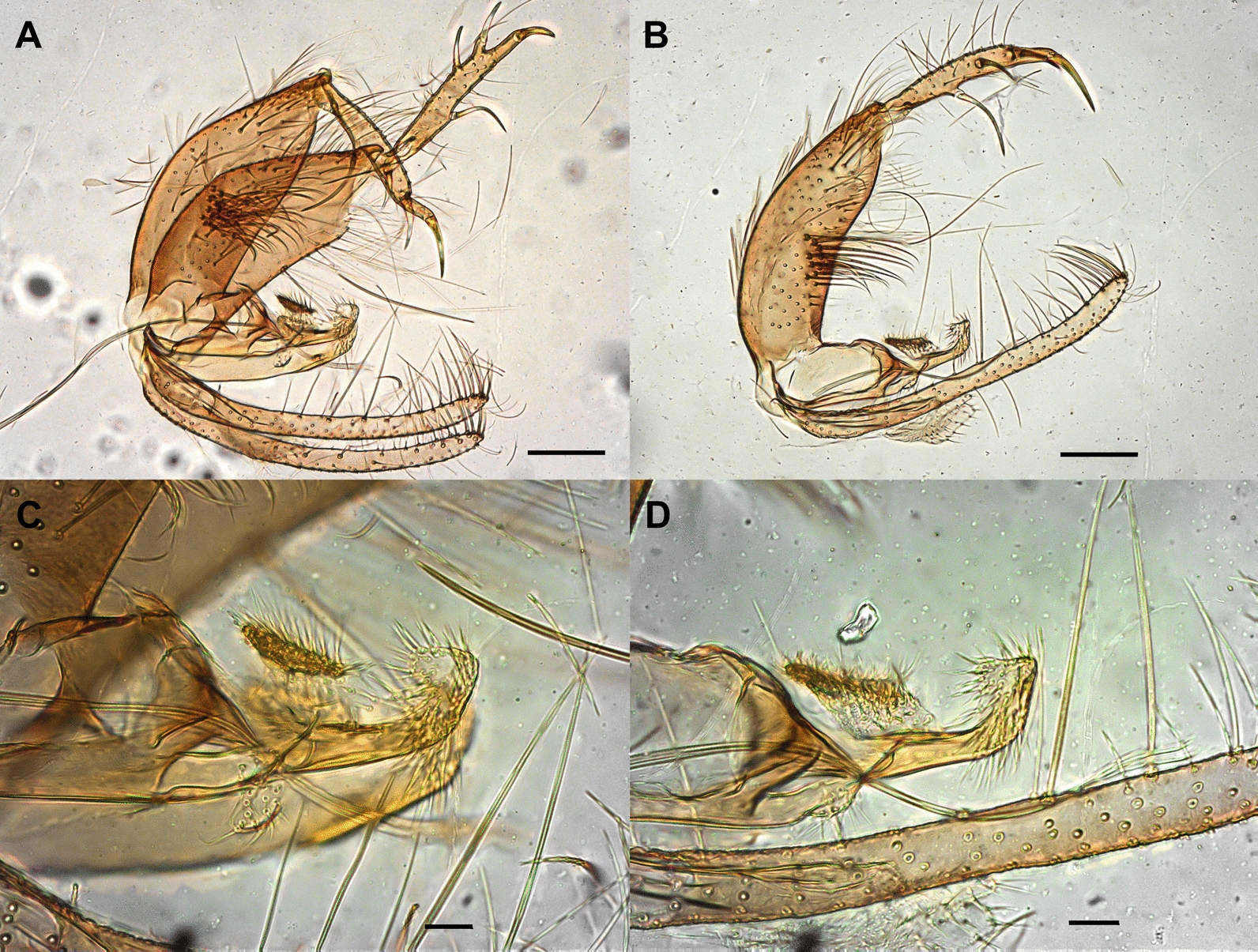
*Trichophoromyia peixotoi* n. sp. holotype (**A** and **C**), and paratype M2 (E-16395) (**B** and **D**): **A**, **B** Male terminalia, lateral view (scale bar: 100 μm). **C**, **D** Paramere, lateral view (scale bar: 20 μm)

**Fig. 5:**
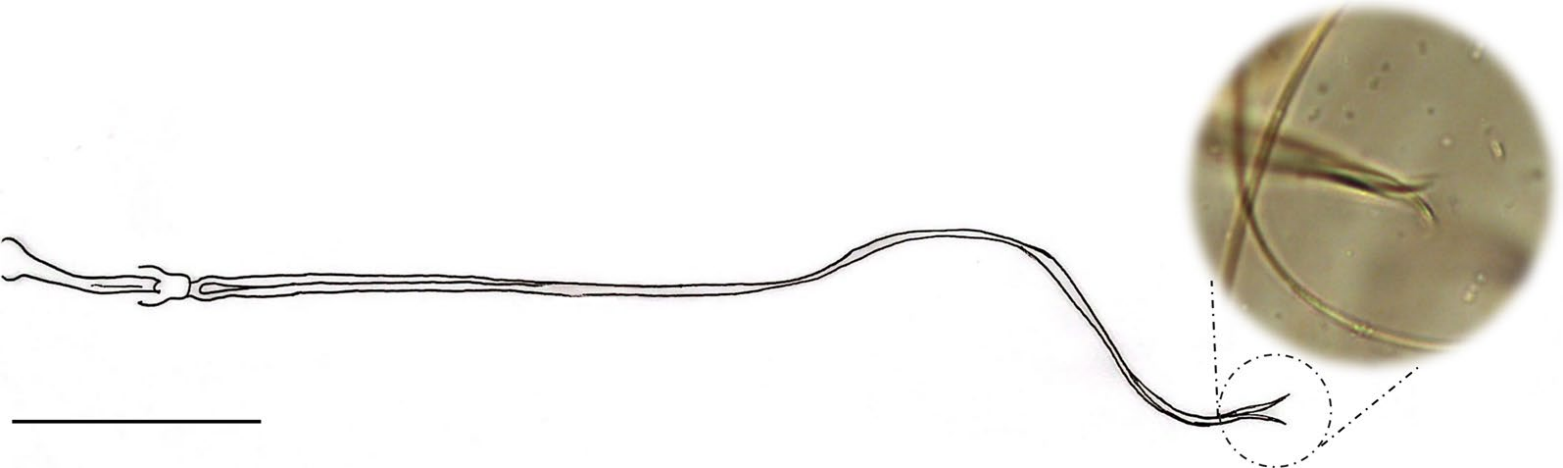
*Trichophoromyia peixotoi* n. sp. holotype. Genital filaments, with a focus on the apex of aedeagal duct (scale bar: 200 μm)

*Trichophoromyia peixotoi* n. sp. Rodrigues, Pinto & Galati (Figs. 1, 2, 3, 4, and 5)

**Diagnosis:** The male of this new species can be distinguished from the others of the genus *Trichophoromyia* by characters of the terminalia, including the paramere with a triangular median lobe in the dorsal margin, and a rectangular paramere apex covered with fine bristles, in addition to the presence in the gonocoxite of a median cluster with about 12 thick and long setae.

### Holotype description (male)

Insect predominantly brown in colour, with the pleurae pale. Pronotum, mesonotum, and metanotum dark brown. Katepisternum and katepimeron brown. Paratergite, upper anepisternum light brown. Medium-sized sand fly, total body length (from the head to the apex of the gonostylus) 2883 (3028.6; 88.71; *n* = 9).

*Head* (Fig. 1): length 373 (364.5; 9.9; *n* = 10), width 333 (331.1; 9; *n* = 9). Clypeus length 104 (100.2; 3.8; *n* = 10). Eye length 212 (206.7; 10.3; *n* = 10), width 101 (103.3; 4.9; *n* = 9). Interocular distance 132 (129.3; 4.3; *n* = 9). Interocular and interantennal sutures unconnected. Flagellomere lengths: FI 242 (222.7; 11; *n* = 9) FII 127 (121.2; 6.5; *n* = 9), FIII 124 (122.2; 4.6; *n* = 9), FXIII 69 (66.3; 2.9; *n* = 9), FXIV 58 (56.6; 4; *n* = 9). Labroepipharynx 221 (212; 8; *n* = 9). Preapical papilla present in FI and FII, but absent in FIII. Presence of apical, median, and basal papillae in FXII–FXIV. Simple setae present in FV–FXIV. Ascoidal formula FI–FXI 2, FXII–FXIV 0; ascoids long with rudimentary posterior spur, and the anterior projection reaching the basis of the subsequent flagellomere; internal/external ascoids implanted at nearly the same level; for FI, the external is slightly more basal than the internal. Labial sutures united. Palpal formula: 1.4.2.3.5. Newstead’s sensilla present in second palpal segment, and scattered in the third palpal segment. Length of the palpal segments: PI 33 (34.9; 3.1; *n* = 10), PII 85 (88.7; 3; *n* = 10), PIII 136 (129.4; 4.2; *n* = 9), PIV 58 (55.7; 3.3; *n* = 9), PV 159 (155.8; 8.7; *n* = 9).

*Cervix*: Ventro-cervical sensilla absent.

*Thorax*: length 525 (538.8; 21.2; *n* = 10), mesonotum length 501 (498.9; 18.8; *n* = 10). Pleurae with four proepimeral setae (3–4; *n* = 10) and 10 upper anepisternal setae (9–15; *n* = 10). Cervical sclerite with two sensilla. Wing (Fig. 2), length 2062 (2058.3; 58.5; *n* = 10), width 561 (573.9; 35.4; *n* = 10). Length of vein sections: R5 1261 (1289; 44.7; *n* = 10), R2 466 (539.4; 51.3; *n* = 10), R2+3 292 (268.9; 19.5; *n* = 10), R2+3+4 244 (238.6; 16.6; *n* = 10), *delta* 298 (365.2; 42.8; *n* = 10), *pi* 181 (150.1; 24.8; *n* = 10). Leg length: anterior, median, and posterior, respectively: coxa 330 (334.6; 11.6; *n* = 10), 319 (331.2; 6.3; *n* = 10), 332 (343.3; 10.9; *n* = 10). The remaining leg segments—femur, tibia, and tarsomeres—are not available for the holotype as they were processed for DNA extraction; however, we provide these measurements for some of the paratypes: femur (811; 32.1; *n* = 3), (784.3; 15.6; *n* = 3), (879.3; 25.5; *n* = 3); tibia (1053.7; 60.3; *n* = 3), (1318.3; 22.2; *n* = 3), (1502.3; 27.7; *n* = 3); tarsomere I (643; 16.8; *n* = 3), (775.3; 22.7; *n* = 3), (844.3; 22; *n* = 3); tarsomeres II+III+IV+V (692; 13; *n* = 3), (756; 28.6; *n* = 3), (824; 20; *n* = 3). Metatarsomere III (two verticils with spines, one median and one apical).

*Abdomen*: length 1394 (1494.7; 80; *n* = 10). Absence of tergal papillae from second to seventh tergites. Terminalia (Figs. 3 and 4): gonocoxite length 365 (382.4; 19.1; *n* = 10), width 124 (132.2; 17.7; *n* = 10); presence of a median cluster with about 12 (10–14; *n* = 10) thick and long setae, and more dispersed and thin bristles in the median-apical region of the gonocoxite, the most apical being thicker than the median portion. Gonostyle 226 (233; 9.8; *n* = 10) long, with four spines, having the following disposition: one apical 72 (85.3; 7; *n* = 10), the upper external preapical 55 (62.8; 8.6; *n* = 10), the lower external implanted after the medium region, so that it is closer to the upper than the internal one, which is implanted at the basal third of the gonostyle. Preapical spiniform setae absent. Paramere simple; dorsal margin length 189 (187.3; 7.8; *n* = 10) forming a triangular median lobe covered with fine bristles; apex of paramere rectangular, covered with fine bristles; ventral margin length of paramere 258 (246.4; 10.2; *n* = 10) with a small cluster of about 12 short setae. Parameral sheath triangular, dorsal margin length 89 (89.6; 5.6; *n* = 10), ventral 52 (55; 5.1; *n* = 10), and basal 72 (76.3; 5.3; *n* = 10). Epandrial lobe length 468 (464.6; 11.5; *n* = 10), width 29 (32; 1.9; *n* = 10). Cercus length 212 (219.2; 13.9; *n* = 10). Sperm pump (Fig. 5) length 172 (171.2; 3.7; *n* = 10); ejaculatory apodeme length 131 (137.1; 5.7; *n* = 10); sperm sac length 56 (52.2; 2.4; *n* = 10), width (26; 26.6; *n* = 10); pavillion width 40 (35.6; 5.5; *n* = 10); aedeagal duct 1020 (1034.2; 66; *n* = 9); ratio of aedeagal duct/sperm pump 5.9 (6; 0.3; *n* = 9); apex of the aedeagal duct tapered (Figs. 4D and 5).

### DNA barcoding

The sequencing and analysis of the *COI* DNA barcoding region resulted in fragments ranging from 584 to 658 base pairs. Of the 10 analysed specimens (one holotype and nine paratypes), *COI* sequences of five paratypes were obtained. The visual inspection of the alignment indicates the absence of stop codons in the middle of sequences, pseudogenes, and/or nuclear copies of mitochondrial origin (NUMT).

In total, the five new *COI* sequences of *T. peixotoi* n. sp. were analysed with 16 publicly available barcode sequences of *T. auraensis* (Mangabeira [33]), *T. octavioi* (Vargas [34]), *T. reburra* (Fairchild & Hertig [35]), and *T. viannamartinsi* (Sherlock & Guitton [36]), totalling an alignment of 21 *COI* sequences and five species (Table 1). The maximum intraspecific K2P distance ranged from 0 to 1.39%, and the minimum distance to the nearest neighbour ranged from 1.92 to 13.3% (Table 1). In general, the interspecific distance was low, but *T. reburra* reached the highest value (13.3%), in discordance with the relationships of the other four species (Table 1).

**Table 1:** DNA barcoded sand flies of the genus *Trichophoromyia*. Number of analysed sequences, maximum intraspecific genetic divergence, and the minimum distance to the nearest neighbour (interspecific) are given in percentage values

Species	*n*	Maximum intraspecific K2P distance (mean)	Distance to the nearest neighbour
*Trichophoromyia auraensis* (Mangabeira)	5	0 (0)	1.92
*Trichophoromyia octavioi* (Vargas)	3	0.86 (0.57)	1.92
*Trichophoromyia peixotoi* n. sp. Rodrigues, Pinto & Galati	5	1.39 (0.79)	1.92
*Trichophoromyia reburra* (Fairchild & Hertig)	3	0.17 (0.11)	13.3
*Trichophoromyia viannamartinsi* (Sherlock & Guitton)	5	0.69 (0.31)	3.89

**Fig. 6:**
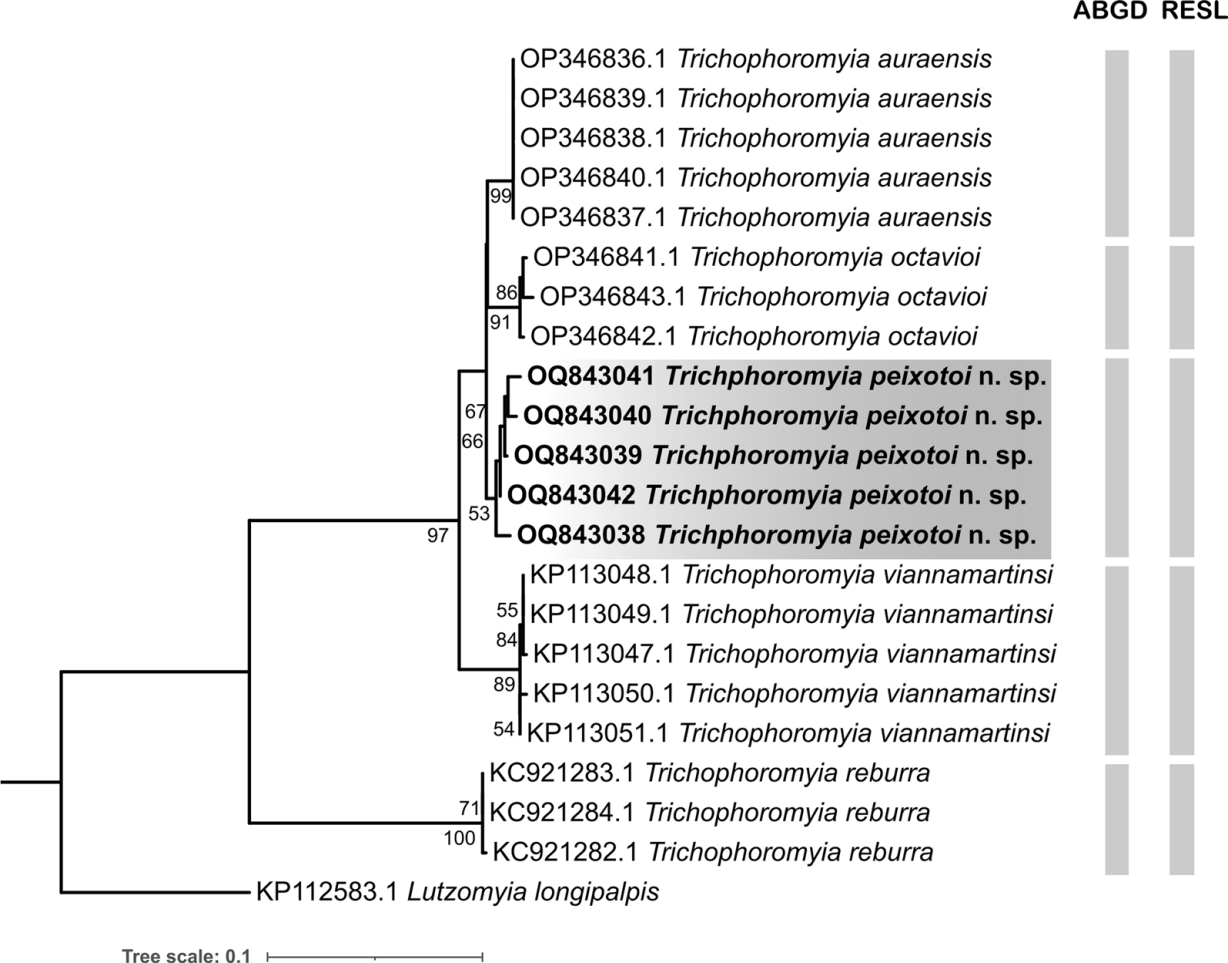
Phylogenetic gene tree based on *COI* DNA barcodes of *Trichophoromyia* spp. Numbers near nodes indicate bootstrap values above 50. Lateral grey bars indicate the MOTU species delimitation partitions made by the algorithms ABGD and RESL

The phylogenetic inference recovered five well-supported clades that are in agreement with the analysed nominal species. In the same sense, the species delimitation algorithms sorted the *COI* sequences in MOTUs that agree with morphological identification (Fig. 6). Also, the ML analysis revealed a single well-supported clade containing the species *T. auraensis*, *T. octavioi*, *T. peixotoi* n. sp., and *T. viannamartinsi*, which seem to be more related to each other than to *T. reburra* (Fig. 6).

### Type material

The male holotype and paratypes were collected in the PNA using CDC-type light traps operated overnight (Pinto et al. Col.). Holotype and three paratypes male were collected on 12 November 2022 near the Uruá base of the PNA. Four paratypes were collected on 7 July 2022, also near the Uruá base, and two were collected on 10 September 2022 near the Tracoá base of the PNA. These two latter specimens were collected in the tree canopy and were slide-mounted using Enecê resin, while the others were above the ground level and mounted using Canada balsam.

The five sand flies processed for molecular analysis correspond to the paratypes which were collected on 10 September 2022 (GenBank accession numbers OQ843041–OQ843042) and 12 November 2022 (OQ843038–OQ843040).

The holotype and paratypes are deposited in the Coleção de Referência da Faculdade de Saúde Pública (FSP–USP) at the Universidade de São Paulo, São Paulo, Brazil under museum numbers E-16393–E-16402.

### ZooBank registration

To comply with the regulations set out in Article 8.5 of the amended 2012 version of the International Code of Zoological Nomenclature [49], details of the new species have been submitted to ZooBank. The Life Science Identifier (LSID) of the article is urn:lsid:zoobank.org:pub:43B5EC2C-239A-4D15-9969-A4C28726C575. The LSID for the new species name *Trichophoromyia peixotoi* is urn:lsid:zoobank.org:act:EF1DD733-4958–4669-BEE8-E35B92788B21.

### Etymology

The name *Trichophoromyia peixotoi* is a tribute to Professor Alexandre Afranio Peixoto, for his great contribution to the knowledge of sand flies, especially in studies on population genetics.

## Discussion

*Trichophoromyia*, a genus of the Psychodopygina subtribe comprising 44 nominal species, is morphologically characterized by the absence of ventro-cervical sensilla and papilla in the third flagellomere, and the presence of Newstead’s sensilla on the second palpal segment; male terminalia equivalent to or longer than the length of the thorax, and short gonostyle apical spine; females having cibarium with more than four pairs of posterior teeth, the anterior teeth distributed in several lines, and a wide sclerotized area; and lacinia with the external teeth implanted in two longitudinal lines and spermathecae with more than 20 rings [8]. The distinction between the species of *Trichophoromyia* has basically been based on the characteristics of the genitalia, especially of males. For females, the great similarity between the spermathecae has been an obstacle to the distinction of species, so that only 50% of them have females described. Among these, *T. reburra* differs from all others of the genus because it presents hyaline concretions on the spermathecal ducts, and *T. cellulana* (Young [37]), *T. omagua* (Martins, Llanos & Silva [38]), *Trichophoromyia* sp. 1 de Araracuara (Morales & Minter [39]), and *T. ubiquitalis* (Mangabeira [33]) are clearly distinguishable from all others by the apical ring of spermathecae which is three or less times as long as the preapical ring, while in the others this ring is six or more times longer [8]. In the present study, together with *T. peixotoi* n. sp. that clearly belongs to *Trichophoromyia* by morphology, we also collected males of *T. ubiquitalis,* and another undescribed *Trichophoromyia* sp. that is being published by another research group. In addition, we collected only female specimens having spermathecae with an apical ring six or more times as long as the preapical. Thus, it was not possible to make a reliable association of sexes by morphological characters, and the female of *T. peixotoi* n. sp. remains unknown.

This new species presents a dorsal lobe covered with thin bristles situated closer to the apex than to the base of the paramere, and the paramere apex facing towards the gonocoxite and gonostyle. These characteristics of the paramere of *T. peixotoi* n. sp. present similarities to those of a group of species constituted by *T. adelsonsouzai* Santos, Silva, Barata, Andrade & Galati [40], *T. brachipyga* (Mangabeira [33]), *T. dunhami* (Causey & Damasceno [41]), *T. eurypyga* (Martins, Falcão & Silva [42]), *T. gibba* (Young & Arias [43]), and *T. viannamartinsi*. However, all these aforementioned species present the cluster of thick bristles of gonocoxite in smaller numbers than *T. peixotoi* n. sp. (10–14), *T. gibba* (4–7), *T. viannamartinsi* (2), and *T. brachipyga* (4–6). In addition, *T. peixotoi* n. sp. also presents clear differences in the shape of the dorsal lobe and apex of the paramere: the dorsal lobe has a more triangular and the apex a more rectangular aspect regarding these closely related species. Moreover, these species have a gonostyle with a short and thicker apical spine, while in *T. peixotoi* n. sp. it is longer and thinner.

Beyond morphology, we accessed the information regarding one mitochondrial molecular marker, the *COI* gene fragment of DNA barcoding. Here, we did not use the *COI* barcode sequences for diagnostic purposes, mainly because most of the described species of the genus *Trichophoromyia* do not have available sequences, with less than 10% of its species processed for the *COI* gene [12], which makes the diagnostic potential of barcodes limited, although molecular analysis of *T. peixotoi* n. sp. supported the description of this new taxon.

Phylogenetic analysis of the *COI* gene recovered the analysed species into single clades each, also in accordance with species delimitation algorithms. Thus, all sequences of *T. peixotoi* n. sp. and the other analysed nominal species have molecular differences that allow the identification between them, including the morphologically similar species *T. viannamartinsi*. This scenario is promising for entomological monitoring, as it would allow the identification of isomorphic females by molecular methods [44–46]. Unfortunately, it was not possible to sequence females of the species collected in the type locality of *T. peixotoi* n. sp., but future studies will be able to associate males and females to elucidate possible morphological differences between females.

Despite the limited number of analysed taxa, *T. peixotoi* n. sp. seems to be closely related to *T. auraensis*, *T. octavioi*, and *T. viannamartinsi*, retrieving a well-supported monophyletic clade. All these species belong to the *Trichophoromyia* group with long aedeagal ducts, which are > 4 times the length of the sperm pump, differing from another group with shorter ducts (≤ 3 times the length of the sperm pump) [8]. The latter comprises *T. reburra*, *T. meirai* (Causey & Damasceno [47]), *T. ubiquitalis*, *T. omagua*, and *T. uniniensis* Ladeia-Andrade, Fé, Sanguinette & Andrade Filho [48]. In our molecular analysis, *T. reburra* had a large genetic divergence compared to the other species of the genus, reaching values of interspecific K2P distances greater than 13%, while the interspecific divergence of the other four *Trichophoromyia* species was always less than 4%. This evidence leads us to suppose that the aedeagal ducts/sperm pump ratio may be a relevant character to sort *Trichophoromyia* species into at least two subgenera, but studies comprising a greater number of taxa and different molecular markers are needed to reinforce this idea.

## Conclusions

The description of *T. peixotoi* n. sp. brings the number of species in this genus to 45, with 24 of them occurring in Brazil. We added new *COI* barcode sequences to DNA repositories, which are useful for *Trichophoromyia* species delimitation, and can be used for further species identification by integrative approaches.

## Data Availability

All sequences obtained from the study were deposited in the GenBank database under the accession numbers OQ843038–OQ843042.

## Abbreviations

*COI*: Cytochrome *c* oxidase subunit I
MOTU: Molecular operational taxonomic unit
ABGD: Automatic barcode gap discovery
RESL: Refined single linkage
ML: Maximum likelihood

## References

[1] Akhoundi M, Kuhls K, Cannet A, Votýpka J, Marty P, Delaunay P, Sereno D (2016). A historical overview of the classification, evolution, and dispersion of *Leishmania* parasites and sandflies. PLoS Negl Trop Dis.

[2] Galati EAB, Rodrigues BL (2023). A review of historical phlebotominae taxonomy (Diptera: Psychodidae). Neotropical Entomol.

[3] Galati EAB (1995). Phylogenetic systematic of the Phlebotominae (Diptera: Psychodidae) with emphasis on American groups. (II Intern. Symp. Phlebotomine Sandflies). Bol de la Dir de Malar Y Saneam Ambiental.

[4] Galati EAB, Andrade-Filho JD, Silva ACL, Falcão AL (2003). Description of a new genus and new species of New World Phlebotominae (Diptera, Psychodidae). Rev Bras de Entomol.

[5] Maroli M, Feliciangeli MD, Bichaud L, Charrel RN, Gradoni L (2013). Phlebotomine sandflies and the spreading of leishmaniases and other diseases of public health concern. Med Vet Entomol.

[6] Santos TV, Silveira FT (2020). Increasing putative vector importance of *Trichophoromyia* phlebotomines (Diptera: Psychodidae). Mem do Inst Oswaldo Cruz.

[7] Aguiar GM, Vieira VR, Elizabeth FR, Jeffrey JS (2018). Regional distribution and habitats of Brazilian phlebotomine species. Brazilian sand flies: biology, taxonomy, medical importance and control.

[8] Galati EAB (2018). Phlebotominae (Diptera, Psychodidae): classification, morphology and terminology of adults and identification of American taxa Brazilian sand flies: biology, taxonomy, medical importance and control.

[9] Sánchez Uzcátegui YD, Santos TV, Póvoa MM (2021). Morphological description of immature stages of *Trichophoromyia brachipyga* (Mangabeira) (Diptera: Psychodidae: Phlebotominae). Zootaxa.

[10] Padial JM, Miralles A, De la Riva I, Vences M (2010). The integrative future of taxonomy. Front Zool.

[11] Depaquit J (2014). Molecular systematics applied to Phlebotomine sandflies: review and perspectives. Infect Genet Evol.

[12] Rodrigues BL, Galati EA (2023). Molecular taxonomy of phlebotomine sand flies (Diptera, Psychodidae) with emphasis on DNA barcoding: a review. Acta Tropica.

[13] Hebert PD, Cywinska A, Ball SL, DeWaard JR (2003). Biological identifications through DNA barcodes. Proceedings of the royal society of London. Series B Biol Sci.

[14] Costa GD, Rocha DD, Júnior AM, Ferreira GE, Medeiros JF, Gonçalves RG, de Andrade AJ (2021). Redescription of two *Psathyromyia* species (Diptera: Psychodidae), including description of the female of *Psathyromyia pradobarrientosi* using molecular and morphological approaches. J Med Entomol.

[15] Sharkey MJ, Janzen DH, Hallwachs W, Chapman EG, Smith MA, Dapkey T, Brown A, Ratnasingham S, Naik S, Manjunath R, Perez K (2021). Minimalist revision and description of 403 new species in 11 subfamilies of Costa Rican braconid parasitoid wasps, including host records for 219 species. ZooKeys.

[16] Meier R, Blaimer BB, Buenaventura E, Hartop E, von Rintelen T, Srivathsan A, Yeo D (2022). A re-analysis of the data in Sharkey et al.’s (2021) minimalist revision reveals that BINs do not deserve names, but BOLD Systems needs a stronger commitment to open science. Cladistics.

[17] Zamani A, Fric ZF, Gante HF, Hopkins T, Orfinger AB, Scherz MD, Bartoňová AS, Pos DD (2022). DNA barcodes on their own are not enough to describe a species. Syst Entomol.

[18] ICMBio (Instituto Chico Mendes de Conservação da Biodiversidade). 2021. Plano de Manejo do Parque Nacional da Amazônia. ICMBio, Itaituba/PA. 53 p.

[19] Forattini, O. P. 1973. Entomologia Médica, Vol. 4. Edgard Blucher, São Paulo, SP.

[20] Cerqueira NL (1943). Novo meio para montagem de pequenos insetos em lâminas. Mem Inst Oswaldo Cruz.

[21] Galati EA, Galvis-Ovallos F, Lawyer P, Léger N, Depaquit J (2017). An illustrated guide for characters and terminology used in descriptions of Phlebotominae (Diptera, Psychodidae). Parasite.

[22] Folmer O, Black M, Hoeh W, Lutz R, Vrijenhoek R (1994). DNA primers for amplification of mitochondrial cytochrome c oxidase subunit I from diverse metazoan invertebrates. Mol Mar Biol Biotech.

[23] Stucky BJ (2012). SeqTrace: a graphical tool for rapidly processing DNA sequencing chromatograms. J Biomol Tech JBT.

[24] Sayers EW, Cavanaugh M, Clark K, Pruitt KD, Schoch CL, Sherry ST, Karsch-Mizrachi I (2022). GenBank. Nucleic Acids Res.

[25] Edgar RC (2004). MUSCLE: multiple sequence alignment with high accuracy and high throughput. Nucleic Acids Res.

[26] Kumar S, Stecher G, Tamura K (2016). MEGA7: molecular evolutionary genetics analysis version 7.0 for bigger datasets. Mol Biol Evol.

[27] Nguyen LT, Schmidt HA, Von Haeseler A, Minh BQ (2015). IQ-TREE: a fast and effective stochastic algorithm for estimating maximum-likelihood phylogenies. Mol Biol Evol.

[28] Trifinopoulos J, Nguyen LT, von Haeseler A, Minh BQ (2016). W-IQ-TREE: a fast online phylogenetic tool for maximum likelihood analysis. Nucleic Acids Res.

[29] Letunic I, Bork P (2021). Interactive tree of life (iTOL) v5: an online tool for phylogenetic tree display and annotation. Nucleic Acids Res.

[30] Blaxter M, Mann J, Chapman T, Thomas F, Whitton C, Floyd R, Abebe E (2005). Defining operational taxonomic units using DNA barcode data. Philos Trans Royal Soc B Biol Sci.

[31] Puillandre N, Lambert A, Brouillet S, Achaz GJ (2012). ABGD, automatic barcode gap discovery for primary species delimitation. Mol Ecol.

[32] Ratnasingham S, Hebert PD (2013). A DNA-based registry for all animal species: the barcode index number (BIN) system. PLoS ONE.

[33] Mangabeira FO (1942). 7ª Contribuição ao estudo dos Flebotomus (Diptera: Psychodidae). Descrição dos machos de 24 novas espécies. Mem do Inst Oswaldo Cruz.

[34] Vargas L (1949). *Phlebotomus octavioi*. Revista del Instituto de Salubridad y Enfermedades.

[35] Fairchild GB, Hertig M (1961). Notes on the *Phlebotomus* of Panama XVI (Diptera, Psychodidae) descriptions of new and little-known species from Panama and Central America. Ann Entomol Soc Am.

[36] Sherlock IA, Guitton N. Notas sobre o subgênero *Trichophoromyia* Barretto, 1961 (Diptera, Psychodidae, Phlebotominae). Revista Brasileira de Biologia. 1970.

[37] Young DG (1979). A review of the blood sucking psychodid flies of Colombia (Diptera, Phlebotominae and Sycoracinae) Revisión de las moscas psicódidas de Colombia (Diptera, Phlebotominae y Sycoracinae). Fla Agric Exp Stn Bull.

[38] Martins AV, Llanos BZ, Silva JE (1976). Estudos sôbre os flebotomíneos do Peru. IV. Departamento de Loreto: lista das espécies coletadas, descrição de uma espécie nova, Lutzomyia omagua n. sp. e redescrição do macho de Lutzomyia scaffi (Damasceno & Arouck, 1956) (Diptera, Psychodidae, Phlebotominae). Revista Brasileira de Biol.

[39] Morales A, Minter DM (1981). Estudio sobre flebotomineos en Araracuara Caquetá, Colombia SA Incluyendo la descripción de *Lutzomyia araracuarensis* (Diptera, Psychodidae). Biomedica.

[40] Santos TV, Silva FM, Barata ID, Andrade AJ, Galati EA (2013). A new species of phlebotomine, *Trichophoromyia adelsonsouzai* (Diptera: Psychodidae) of Brazilian Amazonia. Mem Inst Oswaldo Cruz.

[41] Causey OR, Damasceno RG (1945). Estudo sôbre *Flebotomus* no vale Amazônico: parte II. descrição de *F. dunhami*, *F melloi* e *F. wagleyi* (Diptera, Psychodidae). Memórias do Instituto Oswaldo Cruz.

[42] Martins AV, Falcão AL, Silva JD (1963). Notas sobre os flebótomos do território de Roraima, com a descrição de três novas espécies (Diptera, Psychodidae). Rev Bras Biol.

[43] Young DG, Duncan MA. Guide to the identification and geographic distribution of *Lutzomyia* sand flies in Mexico, the West Indies, Central and South America (Diptera: Psychodidae). Walter Reed Army Institute of Research Washington DC. 1994.

[44] Pinto ID, Chagas BD, Rodrigues AA, Ferreira AL, Rezende HR, Bruno RV, Falqueto A, Andrade-Filho JD, Galati EA, Shimabukuro PH, Brazil RP (2015). DNA barcoding of neotropical sand flies (Diptera, Psychodidae, Phlebotominae): species identification and discovery within Brazil. PLoS ONE.

[45] Kasap EO, Linton YM, Karakus M, Ozbel Y, Alten B (2019). Revision of the species composition and distribution of Turkish sand flies using DNA barcodes. Parasit Vectors.

[46] Lozano-Sardaneta YN, Paternina LE, Sanchez-Montes S, Quintero A, Ibanez-Bernal S, Sanchez-Cordero V, Bejarano EE, Becker I (2020). DNA barcoding and fauna of phlebotomine sand flies (Diptera: Psychodidae: Phlebotominae) from Los Tuxtlas, Veracruz. Mexico Acta Tropica.

[47] Causey OR, Damaceno RG (1945). Estudo sobre *Flebotomus* no vale Amazônico. Parte IV. Descrição de *F. cerqueirai*, *F. dreisbachi*, *F. meirai*, e *F. ferreirai* (Diptera: Psychodidae). Memórias do Instituto Oswaldo Cruz.

[48] Ladeia-Andrade S, Fé NF, Sanguinette CD, Andrade Filho JD (2014). Description of *Trichophoromyia uniniensis*, a new phlebotomine species (Diptera: Psychodidae: Phlebotominae) of Amazonas state. Brazil Parasites Vectors.

[49] ICZN International Commission on Zoological Nomenclature (2012). Amendment of articles 8, 9, 10, 21 and 78 of the International Code of Zoological Nomenclature to expand and refine methods of publication. Bull Zool Nomencl.

